# Bypassing Osmotic Shock Dilemma in a Polystyrene Resin Using the Green Solvent Cyclopentyl methyl Ether (CPME): A Morphological Perspective

**DOI:** 10.3390/polym11050874

**Published:** 2019-05-13

**Authors:** Othman Al Musaimi, Ayman El-Faham, Zainab Almarhoon, Alessandra Basso, Beatriz G. de la Torre, Fernando Albericio

**Affiliations:** 1 Peptide Science Laboratory, School of Chemistry and Physics, University of KwaZulu-Natal, Durban 4001, South Africa; musamiau@gmail.com; 2 KwaZulu-Natal Research Innovation and Sequencing Platform (KRISP), School of Laboratory Medicine and Medical Sciences, College of Health Sciences, University of KwaZulu-Natal, Durban 4041, South Africa; 3 Department of Chemistry, College of Science, King Saud University, P.O. Box 2455, Riyadh 11451, Saudi Arabia; aymanel_faham@hotmail.com (A.E.-F.); zalmarhoon@ksu.edu.sa (Z.A.); 4 Department of Chemistry, Faculty of Science, Alexandria University, P.O. Box 426, Alexandria 21321, Egypt; 5 Purolite, Llantrisant Business Park, Llantrisant, CF72 8LF, UK; alessandra.basso@purolite.com; 6 CIBER-BBN, Networking Centre on Bioengineering, Biomaterials and Nanomedicine, Barcelona Science Park, University of Barcelona, 08028 Barcelona, Spain; 7 Department of Organic Chemistry, University of Barcelona, 08028 Barcelona, Spain

**Keywords:** solid-phase peptide synthesis, osmotic shock, green solvent

## Abstract

The “osmotic shock” phenomenon is the main thing that is responsible for morphological structure alteration, which can jeopardize the use of a polymer in a chemical process. This is extremely important in solid-phase peptide synthesis (SPPS), which is the method of choice for the preparation of these important biologically active compounds. Herein, we have used Hildebrand solubility parameters (δ) to investigate the influence of different ethers that are used in the precipitation step of the SPPS using a polystyrene resin. The green cyclopentyl methyl ether (CPME) has shown to be slightly superior to 2-methyltetrahydrofurane, which is also a green ether and clearly better than the hazardous diethyl ether and *tert*-butyl methyl ether. These results have been corroborated by scanning electron microscope (SEM) analysis and computational studies. All together, these confirm the adequacy of CPME for being the ether of choice to be used in SPPS.

## 1. Introduction

The use of non-soluble polymers is gaining presence in the chemistry arena [1,2]. There is no universal solvent that can be considered as compatible with all of them due to the broad available spectrum of these kinds of polymers. The identification of the most adequate solvent in terms of compatibility and/or swelling could be determined through several parameters. As a result, certain solvents are considered as good solvents, while the others are not (called “bad” solvents). This classification was made mainly based on the “Solubility” parameters of solvents and solutes [3]. In this work, the Hildebrand “Solubility” parameters (δ) will be adopted for analyzing the adequacy of a family of solvents in our current research in green chemistry. This parameter is mainly derived based on the cohesive energy of the chemical compounds [4]. It also considers the addition of other parameters that were introduced by Hansen [5] to account for dispersion forces, polarity, and hydrogen bonding [6]. In summary, materials that have similar or close δ values are likely miscible [7]. The solubility parameters have a great impact on the polymers industry to choose the most convenient solvent. Thus, they help in enhancing the synthetic processes of polymers, where the unreacted monomers could be easily removed, minimizing the by-products and reducing several health and/or environment related concerns [8]. Furthermore, they are playing an enormous role in several applications of the polymer science, such as kidney dialysis, soft contact lenses, and surgical implants, among others [8].

The use of the so-called good solvents, which can be defined as those that show a similar or close δ with the polymer, should preserve its morphological properties. On the other hand, the use of “bad” solvents could cause an alteration in the morphology of the resin. This phenomenon is known as “osmotic shock” phenomenon, which is also observed due to the low mechanical stability of the resin. Osmotic shock could affect the reaction rate as a result of the damaged morphological characteristics of the beads. The mild damage usually appears as a peeling/flaking in the outer shell of the bead, but the bead itself remains intact. Whereas in the higher severity level, the dented tracks become as a major characteristic of the bead, and in the escalated levels dimples and even fractures are most likely taking place.

Crosslinked non-soluble polymeric supports play an important role in the field of heterogeneous reactions [9]. Most importantly, these supports are the base for the solid-phase peptide synthesis (SPPS) [2], which is the method of choice for the synthesis of peptides for both research and industrial purposes. In SPPS, the growing peptide is anchored to the polymeric support, which acts as a kind of protecting group [2,9,10]. In principle, the use of solvents is not limited to a prerequisite of the solubility of these supports in the different solvents used in the reactions, as occurs in the case of homogeneous phase. The solid-phase is compatible with a broad range of solvents. However, the solvents used in SPPS should possess some properties because reactions do not take place in solid state, for instance, the ability to swell the solid support for allowing the reactants to reach the active sites. Furthermore, they must solubilize the soluble reactants (protected amino acids, coupling reagents, deprotection reagents). The presence of crosslinking enables the swelling of the polymer to a certain elastic limit that is imposed by the crosslinks themselves. In some sense, swelling is a kind of solvation process. Swelling is also controlled by the type of solvent in contact with them [11]. At the same time, crosslinking confers a certain degree of mechanical stability to the resin. Therefore, a certain balance should be kept, which assures the mechanical stability as well as the swelling capacity. Furthermore, the crosslinking is also responsible for the easy to handle component of the most part of the polymers that are commercially available due to their uniform particle size distribution and robust spherical shape [12,13]. In summary, the solubility parameters will provide sufficient data for choosing the most adequate solvent for each operation.

To the contrary of what happens with other polymeric process, those that are involved in SPPS could be associated with “Fine Chemistry”. Thus, all of the reactions that are taking place (an amide bond formation, removal of a protecting group) are well defined and should imply almost 100% conversion yields and without side-reaction, which could jeopardize the purification of the final product. Thus, an effective SPPS process should require a strict fine-tuning of solid support, solvents, and reactants looking for the full compatibility of them.

The present work has been carried out using 2-chlorotrityl chloride (CTC) resin as a model [14]. CTC is a polystyrene (PS) based resin, which is the most common in the industrial environment as well as in research. CTC forms 2-chlorotrityl ester with the first amino acid, which allows for the liberation of the peptide while using as low as 1–2% trifluoroacetic (TFA) at the end of the synthetic process. The cleavage of the peptide from the CTC resin with low concentration of TFA renders the full protected peptide, which is able be used in the synthesis of long peptides through the fragment condensation process.

On the other hand, the fully unprotected peptide is obtained when the cleavage is carried out with a higher concentration of TFA (aprox. 90%). Finally, CTC resin is being used as a polymeric protecting group for a rapid and efficient synthesis of unusual protected amino acids by the interchange of protecting groups [15] and for the synthesis of protected dipeptides to be used as the building blocks in more complex SPPS [16]. This last application is fuelled by the convenient commercial price of the CTC resin and, more importantly, for the possibility of being recyclable through a regeneration/activation process.

We would like to point out that this study was also carried out on two other PS resins that were tested in our laboratory “[Aminomethyl (AM), 4-methylbenzhydrylamine (MBHA)]”, and the results were comparable with those for the CTC resin (Supplemental Figure S1A,B). However, here we focused on CTC resin due to its regeneration capability. The similarity among the three PS resins tested basically indicates that the linkers do not play a role in the final morphology of the resin or the way it is being affected to the introduced solvents, and the main role is basically only ascribed to the PS part.

It is worth noting that the reaction kinetics in SPPS is diffusion-dependent, where the diffusion of the reactants, reagents, and solvents first takes place inside of the gel type beads, followed by excluding them to accommodate the growing molecule [9,15,16,17,18,19,20]. This means that each bead represents a reaction vessel. Thus, the morphological characteristics of those beads must be analyzed through the whole synthetic process. Otherwise, it will have similar effects to using a broken reaction vessel in a conventional solution synthesis.

**Figure 1:**
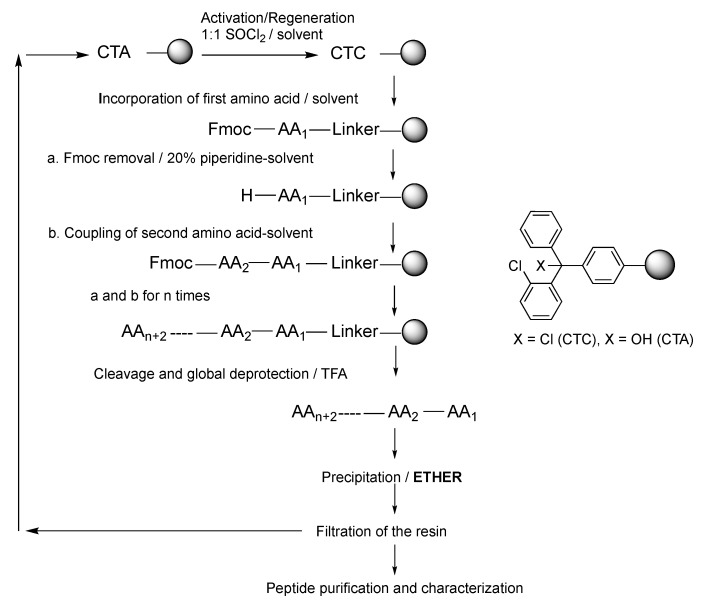
Schematic representation of the solid-phase peptide synthesis (SPPS) and the solvents.

Figure 1 shows a schematic representation of the different steps existing in a SPPS strategy while using CTC resin. First of all, the native resin should be swollen, followed by the incorporation of the first amino acid, removal of the temporary protecting group, elongation of the peptide, and ending with the final deprotection and cleavage from the resin. Furthermore, the washings are being carried out after each step with an appropriate solvent [2]. When CTC or other resin is used for the synthesis of unprotected peptides, these are usually precipitated in the reaction medium with an ether. Ether washing is also introduced in several protocols for removing impurities from the resin and/or before drying the resin [21]. Finally, the 2-chlorotrityl alcohol (CTA) resin that was obtained after the cleavage step could be reused post-reactivation with SOCl_2_ (Figure 1) [15].

While taking into account that ethers are the most disruptive solvents that are used in the SPPS strategy, we report the influence of ethers incorporated in the precipitation step of SPPS on the morphological structure of CTC resin: diethyl ether (DEE) [22], *tert*-butyl methyl ether (MTBE) [22], cyclopentyl methyl ether (CPME) [23], and 2-methyltetrahydrofuran (2-MeTHF) [24]. The ethers are used during the precipitation of the final peptide after global deprotection, for washings, and for facilitating the dryness. CTC resins after the treatment with those ethers have been monitored by a scanning electron microscope (SEM). Finally, computational studies have been carried for better understanding and interpreting the experimental findings.

DEE and MTBE were traditionally used in SPPS, but they have been classified as hazardous chemicals [25,26]. Therefore, the corresponding authorities have recommended their replacement by safer alternatives [26,27,28]. In addition, MTBE shows instability in acidic conditions and provokes undesired *tert*-butylation reactions of the cleaved peptide, which influences the purity of the final product [22]. In this context, our group has recently recommended the use of the green CPME and 2-MeTHF as the replacing ethers for DEE and MTBE [23,24].

## 2. Experimental

PuroSynth CTC (1.0 mmol/g, supplier’s specification) resin was used for all of the syntheses. All the reagents and solvents were obtained from commercial suppliers and were used without further purification, unless otherwise stated.

### 2.1. First Amino acid Incorporation Procedure

CTC resin was swollen in CH_2_Cl_2_ for 15 min. The 9-fluorenylmethyloxycarbonyl (Fmoc)-amino acids (1.2 equiv.) were dissolved in a minimum amount of CH_2_Cl_2_ (0.5 mL/100 mg resin) and sonicated for 10 min. Diisopropylethylamine (DIEA) (4 equiv.) was then added to the solution, and the final solution was then added to the swollen resin and allowed to react for 1 h under mechanical shaking. As soon as the 1 h shaking ends, CH_3_OH was added to the reaction mixture (80 μL/100 mg resin) in order to endcap any of the unreacted chloride of the CTC resin. Finally, the resin was washed twice with 2-MeTHF and dried under vacuum.

### 2.2. Peptide Elongation Procedure

The peptides were synthesized following the standard Fmoc/*t*Bu strategy that was performed in our laboratory; 3 equiv. Fmoc-AA-OH, 3 equiv. OxymaPure, 3 equiv. *N,N*-diisopropylcarbodiimide (DIC) in *N,N*-dimethylformamide (DMF) and then shaking for 1 h. Fmoc was then removed while using 20% piperidine-DMF [23,29,30].

### 2.3. Precipitation Procedure

The synthesized peptides were cleaved from the resin using TFA: triisopropylsilane (TIS): H_2_O (95:2.5:2.5) (1 mL/100 mg resin) under mechanical shaking for 1 h. The same amount of each chilled ether was then added (five times the cleavage solution volume) and the solution (containing the cleaved peptide and the resin) was kept in an ice bath for 30 min. The solution was then centrifuged for 5 min. at 5000 rpm, and the supernatant was decanted. A new amount of each ether (five times the cleavage solution volume) was added to repeat this step. The solution was decanted and any remaining ether was dried under N_2_. An appropriate solvent was added to dissolve the synthesized peptide. Finally, the peptide solution was filtered and the resin was dried under vacuum.

### 2.4. Activation Procedure

The cleaved resin was washed three times with CH_2_Cl_2_, and then a mixture of 1:1 SOCl_2_: CH_2_Cl_2_ was added to cover the whole resin and allowed to react for 4 h under mechanical shaking. After the 4 h shaking ends, the resin was filtered and washed six times with CH_2_Cl_2_. The same procedure is also used for the native resin.

### 2.5. SEM Analysis

The dried resin beads (of each stage mentioned in the manuscript) were coated with a thin layer of gold using a Quorum Q150 RES sputter coater (Quorum Technologies Ltd, East Sussex, UK) and photographed using a scanning electron microscope where the electron beam was set to 5.00–6.00 kV (Zeiss ultra plus FE-SEM, Jena, Germany) to characterize the surface morphology.

## 3. Results

Table 1 shows the solubility parameters that are associated with the 1% DVB crosslinked PS and each ether used in the precipitation step after cleavage.

**Figure 2:**
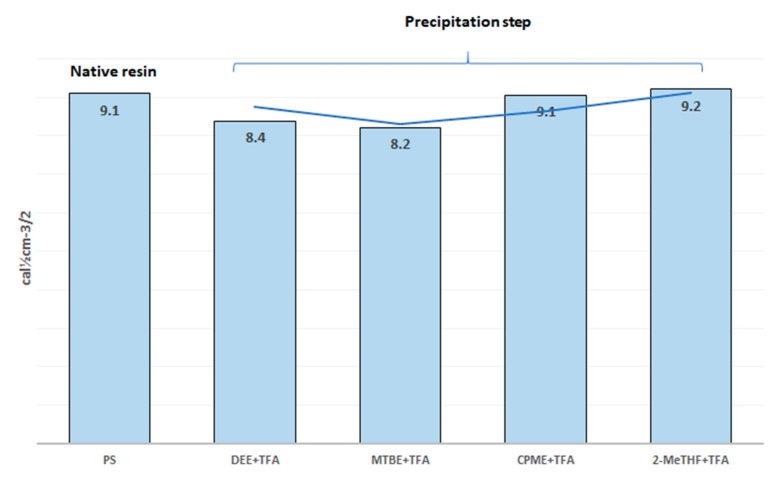
Solubility parameters of PS and ethers used in SPPS protocol. Solubility parameter of each ether was revised based on the ratio of each mixture, [ether-trifluoroacetic (TFA) (5:1)].

It should be noted that the solubility parameter values are additive components [36]. Thus, the solubility parameter value of the ethers was revised based on the percentage of the ether in the final mixture [ether-TFA (5:1)] (Figure 2). It is important to highlight that, when the ethers are considered alone (Table 1), the solubility parameters diverge more from the corresponding of PS.

SEM was used to monitor the morphological structure of the CTC resin at several stages: (i) before starting the synthetic process (native resin); (ii) after the synthetic process (protected peptide anchored on the resin); (iii) after the cleavage step and the addition of the chilled precipitating ether (resin and the cleaved peptide); and, (iv) after the regeneration step. SEM analyses are interpreted in terms of the solubility parameter of 1% divinylbenzene (DVB) crosslinked PS and the corresponding ethers, in which, the values must be compared to estimate the resin-solvent compatibility and interpret the SEM data accordingly.

The dried resin beads of each mentioned stage were coated with a thin layer of gold using Quorum Q150 RES sputter coater (Quorum Technologies Ltd, East Sussex, UK), and photographed using SEM, where the electron beam was set to 5.00–6.00 kV (Zeiss ultra plus FE-SEM, Jena, Germany). The SEM images proved that the full elongation process itself has no effect on the morphology of the beads. Thus, the morphological characteristics of the beads before the cleavage step was similar to the native resin before the synthetic process starts (Supplemental Figure S2A,B).

CTA resin (with no previous syntheses) was regenerated/activated and the SEM images have also proved the absence of the osmotic shock phenomenon. Thus, the morphological properties of its beads were not affected (Supplemental Figure S2C), which is apparently attributed to the closeness between the solubility parameters of the activation solution (SOCl_2_ in dichloromethane (CH_2_Cl_2_), 1:1) (9.6 cal^1/2^cm^−3/2^) [6] and that of the resin (9.1 cal^1/2^cm^−3/2^) [9,31].

From a theoretical point of view and based on the solubility parameters values (Figure 2), CPME and 2-MeTHF could be considered as good solvents of PS (CTC) resin due to their close solubility parameters (9.1, 9.2 and 9.1 cal^1/2^cm^−3/2^, respectively). On the other hand, DEE (8.4 cal^1/2^cm^−3/2^) and MTBE (8.2 cal^1/2^cm^−3/2^) are considered to be “bad” solvents of CTC resin due to the high differences in their solubility parameters. Accordingly, the chance of osmotic shock occurring upon the introduction of DEE or MTBE as precipitating ethers is expected to be higher than in the case with CPME or 2-MeTHF.

**Figure 3:**
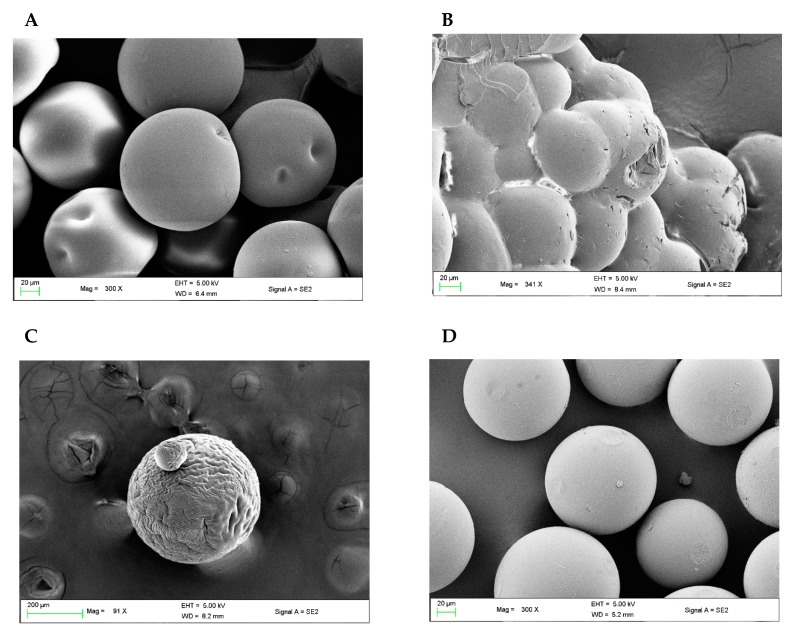
Scanning electron microscope (SEM) images of 2-chlorotrityl chloride (CTC) resin: (**A**) diethyl ether (DEE) (dimples); (**B**) tert-butyl methyl ether (MTBE) (fracture); (**C**) 2-methyltetrahydrofuran (2-MeTHF) (dented tracks); and, (**D**) cyclopentyl methyl ether (CPME) (intact).

The SEM images have shown that CTC resin has suffered from the osmotic shock to different extents upon treating it with three of the precipitating ethers used in this study: DEE, MTBE (Figure 3A,B), and, surprisingly, 2-MeTHF (Figure 3C).

Osmotic shock results when polymer chains gather as a result of restoring their intermolecular stickiness [12], which appears as cracking and sometimes fracturing of the resin’s beads [9,37]. As a result of a gap in the solubility parameters of DEE and/or MTBE with that of CTC resin, severe morphological damage was observed when introducing any of the ethers in the final precipitation step. In the case of DEE, clear dimples were observed in the resin’s beads (Figure 3A). Whereas, a fracture of the beads was observed in the case of MTBE (Figure 3B). 2-MeTHF, which has a close solubility parameter to that of CTC resin, was not expected to affect the morphological structure of the CTC resin, just like CPME; however, unexpectedly it did affect the morphological structure of CTC resin, but to a lower extent than DEE and MTBE. Thus, the resin did not experience dimples or fractures in its beads; in contrast, only dented tracks were observed, which is considered to be a mild effect (Figure 3C). However, this unexpected behavior of 2-MeTHF was explained by a computational study (see the corresponding section).

Contrary to the previously mentioned three ethers, CPME has proven to be an optimum ether and compatible with the PS resins for the precipitation of peptides after the final global deprotection step and for washings/dryings during the synthesis. Thus, no osmotic shock was observed, and the morphological structure of the CTC resin remained intact after the treatments (Figure 3D). After treatment with CPME, the cleaved CTC resin was regenerated using the activation mixture (thionyl chloride- CH_2_Cl_2_, 1:1), and the SEM images also proved that the morphological structure of its beads remained intact (Supplemental Figure S2D), which means that the resin is now ready for new syntheses to be carried out.

### 3.1. Computational Study

Computational chemistry is a unique tool for predicting, corroborating and/or understanding some of the experimental data. In this regard, to explain the unexpected effect of 2-MeTHF on the CTC resin, several computational tests were performed.

Computational studies were carried out using BIOVIA Materials Studio 2016, Accelrys Inc. (San Diego, CA, USA).

As discussed earlier, the linker does not have any influence on the morphological structure; 1% DVB crosslinked PS polymer was considered in this work to represent CTC resin.

**Figure 4:**
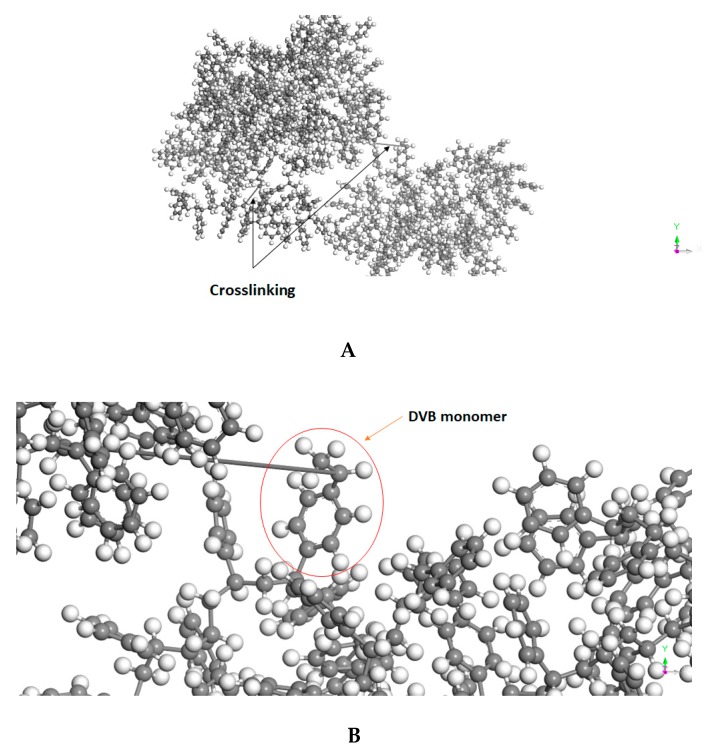
Three-dimensional (3D) Atomistic structure of 1% divinylbenzene (DVB) crosslinked polystyrene (PS) showing: (**A**) the locations of crosslinks; and, (**B**) the DVB monomer.

PS was computationally generated using a PS assemble that was already supported with the software. Subsequently, two monomer units were converted to DVB, followed by crosslinking them to the polymeric chain (Figure 4A). The final structure comprises 1% DVB (Figure 4B) and the final molecular weight of the crosslinked polymer is 21,930 g/mol. The ether assembles were generated using simple atomistic representation in the materials studio interface.

All of the generated assemblies were energy minimized and the geometry was optimized using the “Forcite module” when considering the smart algorithms, where the forcefield was set to “Dreiding” and the charges to “Charge using QEq”. Swelling capacities were simulated using “Blends module”, where the forcefield and charges were set to match those that were selected in the geometry optimization settings. Subsequently, the calculations option was set to “mixing” in order to predict the following three values: mixing energy (Emix), interaction energy, and Chi (χ) parameter values.

The polymer-solvent interaction parameter χ value and Emix value computationally obtained reflects the degree of miscibility/swelling capacity between the solutes and the solvents, where the more the χ and/or Emix values increase, the less miscible/swelling capacity the pair contains, and vice versa.

### 3.2. Computational Data

Examining the obtained χ and Emix values from computational analyses (Table 2) lead us to observe the following swelling capacity trends for the four ethers that were incorporated in this study: CPME > MTBE > 2-MeTHF > DEE. It was observed that the Emix and χ values of 2-MeTHF lie in the region of the “bad” solvents, next to DEE, and even higher than the MTBE. This could explain the unexpected behaviour of 2-MeTHF and the incompatibility of PS resin towards this ether, especially after a SPPS protocol. These results agree with the fact that the osmotic shock did happen, and the morphological structure of the resin was altered and appeared as dented tracks characteristics (Figure 3C). For the rest of the ethers that were used in this study, CPME has the lowest Emix and χ values, which again explains the great performance and compatibility of this green ether over the others, whereas DEE occupies the highest Emix and χ values, hence it was the worst ether in this study. MTBE values are smaller than 2-MeTHF and closer to CPME; however, due to its dissimilar solubility parameter, it is considered as a “bad” solvent was previously realized from the experimental data.

To better understanding the behavior of the ethers, additional computational analyses were carried out. For instance, the dependence of the χ and Emix parameters on the temperature was estimated as a function of temperature when considering 25 steps covering (50–500 K). The calculations showed that, for all of the examined ethers, the lower χ values (good swelling capacity) are likely associated with a temperature as high as 400 K and more; however, such high temperatures do not apply to our experimental conditions, alternatively, as low as 280 K is considered in our experimental procedure. As the temperature goes down, the situation was the opposite, in which higher χ values (worse swelling) are likely more favoured. However, and surprisingly, CPME and MTBE have shown an exceptional trend in a temperature range starting from 270 K to as low as 86 K, where low χ values (good swelling capacity) with these two ethers are also anticipated in the case of temperature reduction. However, at temperatures below 86 K, the normal trend of high χ values is operating again; nevertheless, this exceptional trend is advantageous in our work, as it is operating in a range that covers the working temperature of our study.

**Figure 5:**
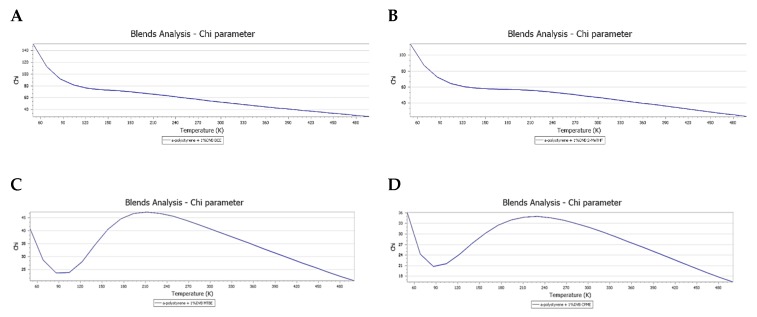
Chart displaying Chi (χ) dependence on temperature (50–500 K): (**A**) DEE; (**B**). 2-MeTHF; (**C**) MTBE; and, (**D**) CPME. Chi (χ) values at the working temperature in this study (280 K): DEE; 55.5 > 2-MeTHF; 49.3 > MTBE; 42.8 > CPME; 33.2.

These calculations explain the experimental data of all the ethers examined in this study, most importantly, the situation of 2-MeTHF. Provided that, for the sake of enhancing the precipitation process, a chilled ether is usually considered rather than a warm one, where the precipitation step is usually carried out at −18 °C (280 K). Thus, the computational data are in line with the experimental findings that consider DEE and 2-MeTHF as “bad” solvents, because they possess high χ values at low temperatures (Figure 5A,B). Despite the fact that MTBE is also showing an interesting reduction trend of χ values as the temperature goes as low as 86 (Figure 5C), it is considered to be “bad” solvent due to its overall high χ values in addition to its dissimilar solubility parameter. This was also experimentally confirmed. On the other hand, CPME was proved to be an optimum ether of choice ascribed to its lowest χ values in comparison to the other three ethers throughout the entire examined temperature range (50–500 K), provided that it shares low χ values, even in the case of temperature reduction, as low as 86 K (Figure 5D). Thus, supporting its optimum compatibility with the CTC resin, which has translated in bypassing osmotic shock and reserving the morphological characteristics of its beads.

**Figure 6:**
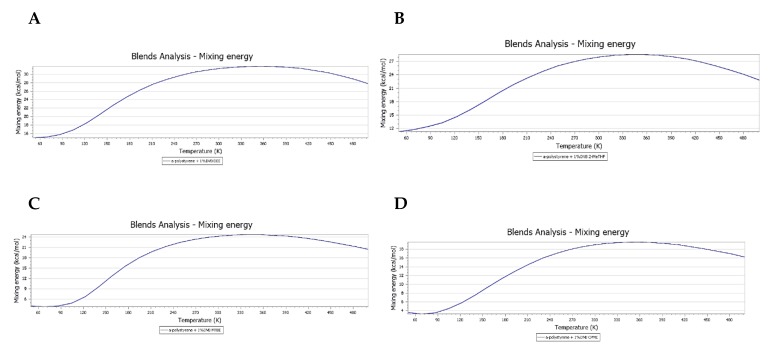
Chart displaying Emix dependence on temperature (50–500 K): (**A**) DEE; (**B**) 2-MeTHF; (**C**) MTBE; and, (**D**) CPME. Emix values at the working temperature in this study (280 K): DEE; 30.9 > 2-MeTHF; 27.4 > MTBE; 23.8 > CPME; 18.5.

Furthermore, analysis of Emix charts also showed the CPME has the lowest Emix values and, hence, the best swelling capacity over the entire examined temperature range (Figure 6A–D).

**Figure 7:**
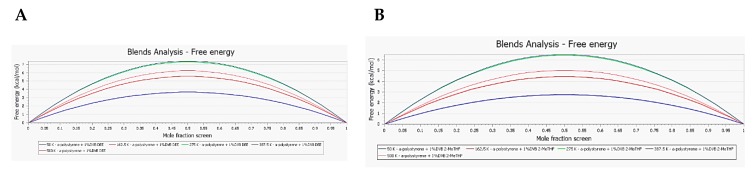

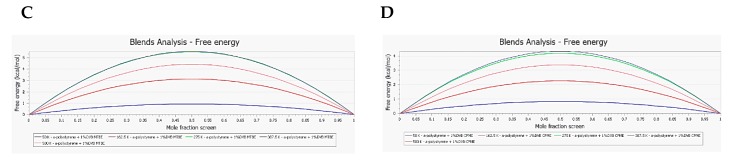
Chart displaying free energy dependence on temperature (50–500 K): (**A**) DEE; (**B**) 2-MeTHF; (**C**) MTBE; and, (**D**) CPME. Swelling free energy in Kcal/mol at 280 K and 0.5 mole fraction: DEE 7.4 > 2-MeTHF 6.5 > MTBE 5.6 > CPME 4.2.

Secondly, an analysis of the swelling free energy dependence on the temperature was performed over the range (50–500 K). As the case with χ and Emix relationship with the temperature, it was also observed that CPME has occupied the lowest swelling free energy values over the examined temperature range, which indicates that this ether is thermodynamically the most favored one among the others analyzed in this study (Figure 7A–D). Furthermore, in the case of CPME, the swelling free energy at the working temperature (280 K) is 4.2 Kcal/mol, which is the lowest value among the ethers. However, it went up to 7.4 Kcal/mol in the case of DEE. The swelling free energy has provided an additional explanation for the unexpected behaviour of 2-MeTHF. Its swelling free energy is 6.5 Kcal/mol, which is considered to be a high value and less thermodynamically favoured. In addition, this value is even higher than that of MTBE (5.6 Kcal/mole), as was also observed in the trend of its χ and Emix values. In conclusion, the swelling free energy data are in line with the experimental findings and they have provided an explanation of the excellent performance of CPME, as well as the observed adverse effect of the 2-MeTHF on the morphological characteristics of CTC resin and the high chance of osmotic shock occurring.

## 4. Conclusions

CPME is a green ether suitable for the precipitation step after the final global cleavage step [23]. Herein, we have also proved the suitability of this green ether from a morphological perspective. We utilized SEM and computational tools to consolidate our findings. The morphological characteristics of CTC resin were intact upon introducing CPME in the precipitation step, in which no osmotic shock phenomenon was observed. At the beginning of the study, we were expecting 2-MeTHF, which is also green ether, to also be suitable for CTC resin (due to its close solubility parameter with PS). However, unfortunately, the experimental results have shown the opposite.

The use of this ether in the precipitation step has provoked the osmotic shock phenomenon in the CTC resin. However, this effect has taken place to a lesser extent than when DEE or MTBE are used.

As a final conclusion, we can say that the green ether CPME has previously proved to be a good precipitating ether in SPPS from just a chemical point of view [23] and, in this work, we have also showed that CPME does not raise osmotic shock. These two properties enhance the opportunity of recycling CTC resin that is to be used in further syntheses. This is also considered to be a green strategy [38], which reinforces our commitment in greening the SPPS.

## Figures and Tables

**Table 1:** Solubility parameters of 1% DVB crosslinked polystyrene (PS) and the ethers used in this study.

Entry	Resin/Solvent	Solubility Parameter (cal^1/2^ cm^−3/2^)
1	1% DVB crosslinked PS	9.1 [9,31]
2	TFA	12.5 [32]
3	DEE	7.6 [7]
4	CPME	8.4 [33]
5	MTBE	7.4 [34]
6	2-MeTHF	8.5 [33,35]

**Table 2:** Emix and Chi (χ) values of each ether and the CTC resin.

Parameter/Solvent	DEE	2-MeTHF	MTBE	CPME
Emix kcal/mol (at 280 K)	30.9	27.4	23.8	18.5
Chi (χ) (at 280 K)	55.5	49.3	42.8	33.2

Blends module, Base: CTC resin; Screen: ether. Geometry optimization done by the Forcite module. “Dreiding forcefield” and “Charge using QEq” were chosen in both of the tests.

## Supplementary Materials

The following are available online at https://www.mdpi.com/2073-4360/11/5/874/s1, Figure S1: Scanning electron microscope (SEM) images of: A. Aminomethyl (AM) resin; B. 4-methylbenzhydrylamine (MBHA) resin Figure S2: Scanning electron microscope (SEM) images of 2-chlorotrityl chloride (CTC) resin.

## References

[1] Rivas B.L., Urbano B.F., Sanchez J. (2018). Water-soluble and insoluble polymers, nanoparticles, nanocomposites and hybrids with ability to remove hazardous inorganic pollutants in water. Front Chem.

[2] Merrifield R.B. (1963). Solid phase peptide synthesis. I. The synthesis of a tetrapeptide’. J. Am. Chem. Soc..

[3] Hildebrand J.H. (1916). Solubility. J. Am. Chem. Soc..

[4] Lee S.H., Lee S.B. (2005). The hildebrand solubility parameters, cohesive energy densities and internal energies of 1-alkyl-3-methylimidazolium-based room temperature ionic liquids. Chem. Commun. (Camb).

[5] Hansen C.M. (2007). Hansen solubility parameters: A user’s handbook..

[6] Wypych A., Wypych G. (2015). Biocides included in article 95 list. Databook of Biocides.

[7] Hansen C.M. The three dimensional solubility parameter and solvent diffusion coefficient, their importance in surface coating formulation. https://www.hansen-solubility.com/contents/HSP1967-OCR.pdf.

[8] Miller-Chou B.A., Koenig J.L. (2003). A review of polymer dissolution. Prog. Polym. Sci..

[9] Sherrington D.C. (1998). Preparation, structure and morphology of polymer supports. Chem. Commun..

[10] Vaino A.R., Janda K.D. (2000). Solid-phase organic synthesis: A critical understanding of the resin. J. Comb. Chem..

[11] Belmares M., Blanco M., Goddard W.A., Ross R.B., Caldwell G., Chou S.H., Pham J., Olofson P.M., Thomas C. (2004). Hildebrand and hansen solubility parameters from molecular dynamics with applications to electronic nose polymer sensors. J. Comput. Chem..

[12] Kangwansupamonkon W., Damronglerd S., Kiatkamjornwong S. (2002). Effects of the crosslinking agent and diluents on bead properties of styrene-divinylbenzene copolymers. J. Appl. Polym. Sci..

[13] Yang W., Ming W., Hu J., Lu X., Fu S. (1998). Morphological investigations of crosslinked polystyrene microspheres by seeded polymerization. Colloid. Polym. Sci..

[14] Athanassopoulos P., Barlos K., Gatos D., Hatzi O., Tzavara C. (1995). Application of 2-chlorotrityl chloride in convergent peptide synthesis. Tetrahedron Lett..

[15] Fields G.B., Field C.G. (1991). Solvation effects in solid-phase peptide synthesis. J. Am. Chem. Soc..

[16] Pugh K.C., York E.J., Stewart J.M. (1992). Effects of resin swelling and substitution on solid phase synthesis. Int. J. Peptide Protein Res..

[17] Krchnak V., Flegelova Z., Vagner J. (1993). Aggregation of resin-bound peptides during solid-phase peptide synthesis. Prediction of difficult sequences Int. J. Peptide Protein Res..

[18] Tam J.P., Lu Y.-A. (1995). Coupling difficulty associated with interchain clustering and phase transition in solid phase peptide synthesis. J. Am. Chem. Soc..

[19] Palomo J.M. (2014). Solid-phase peptide synthesis: An overview focused on the preparation of biologically relevant peptides. RSC Adv..

[20] Labadie J.W. (1998). Polymeric supports for solid phase synthesis. Curr. Opin. Chem. Biol..

[21] Renil M., Pillai V.N.R., Thennarasu S., Nagaraj R. Solid-Phase Peptide Synthesis Using a New Ps-Ttegda Resin: Synthesis of Pardaxin (1-26). http://nopr.niscair.res.in/bitstream/123456789/16623/1/IJCB%2038B%289%29%201030-1035.pdf.

[22] de La Torre B.G., Andreu D. (2008). On choosing the right ether for peptide precipitation after acid cleavage. J. Pept. Sci..

[23] Al Musaimi O., Jad Y.E., Kumar A., Collins J.M., Basso A., de la Torre B.G., Albericio F. (2018). Investigating green ethers for the precipitation of peptides after global deprotection in solid-phase peptide synthesis. Curr. Opin. Green Sustain. Chem..

[24] Al Musaimi O., Jad Y.E., Kumar A., El-Faham A., Collins J.M., Basso A., Torre B.G.d.l., Albericio F. (2018). Greening the solid-phase peptide synthesis process. 2-methf for the incorporation of the first amino acid and precipitation of peptides after global deprotection. Org. Process Res. Dev..

[25] Prat D., Pardigon O., Flemming H.-W., Letestu S., Ducandas V., Isnard P., Guntrum E., Senac T., Ruisseau S., Cruciani P., Hosek P. (2013). Sanofi’s solvent selection guide: A step toward more sustainable processes. Org. Process Res. Dev..

[26] Alder C.M., Hayler J.D., Henderson R.K., Redman A.M., Shukla L., Shuster L.E., Sneddon H.F. (2016). Updating and further expanding gsk’s solvent sustainability guide. Green Chem..

[27] Prat D., Hayler J., Wells A. (2012). A survey of solvent selection guides. Green Chem..

[28] Henderson R.K., Jiménez-González C., Constable D.J.C., Alston S.R., Inglis G.G.A., Fisher G., Sherwood J., Binks S.P., Curzons A.D. (2011). Expanding gsk’s solvent selection guide—embedding sustainability into solvent selection starting at medicinal chemistry. Green Chem..

[29] Jad Y.E., Acosta G.A., Khattab S.N., de la Torre B.G., Govender T., Kruger H.G., El-Faham A., Albericio F. (2016). 2-methyltetrahydrofuran and cyclopentyl methyl ether for green solid-phase peptide synthesis. Amino Acids.

[30] Kumar A., Jad Y.E., Collins J.M., Albericio F., de la Torre B. (2018). Microwave assisted green solid-phase peptide synthesis using γ-valerolactone (gvl) as solvent. ACS Sustain. Chem. Eng..

[31] Rabelo D., Coutinho F.M.B. (1994). Structure and properties of styrene-divinylbenzene copolymers. Polym. Bull..

[32] Marciniak A. (2010). The solubility parameters of ionic liquids. Int. J. Mol. Sci..

[33] Watanabe K., Yamagiwa N., Torisawa Y. (2007). Cyclopentyl methyl ether as a new and alternative process solvent. Org. Process Res. Dev..

[34] Methyl t-butyl ether (mtbe). https://monumentchemical.com/uploads/files/TDS/MTBE%20-%20TDS.pdf.

[35] Aycock D.F. (2007). Solvent applications of 2-methyltetrahydrofuran in organometallic and biphasic reactions. Org. Process Res. Dev..

[36] Burke J. (1984). Solubility Parameters: Theory and Application.

[37] Meldal M. (1997). Properties of solid supports. Methods Enzymol..

[38] Anastas P.T., Zimmerman J.B. (2003). Through the 12 principles green engineering. Environ. Sci. Technol..

