# Supplementary material for: Bypassing Osmotic Shock Dilemma in a Polystyrene Resin Using the Green Solvent Cyclopentyl methyl Ether (CPME): A Morphological Perspective
Source: Polymers (Basel). 2019 May 13;11(5):874. doi: 10.3390/polym11050874 (PMC6571811; doi:10.3390/polym11050874)
Supplement: Supplementary file 1 [file polymers-11-00874-s001.pdf]

*Polymers* **2019**, *11*, 874; doi:10.3390/polym11050874 

# Supplementary Materials: Bypassing Osmotic Shock Dilemma in a Polystyrene Resin Using the Green Solvent Cyclopentyl methyl Ether (CPME): A Morphological Perspective

**Othman Al Musaimi, Ayman El-Faham, Zainab Almarhoon, Alessandra Basso, Beatriz G. de la Torre, and Fernando Albericio**

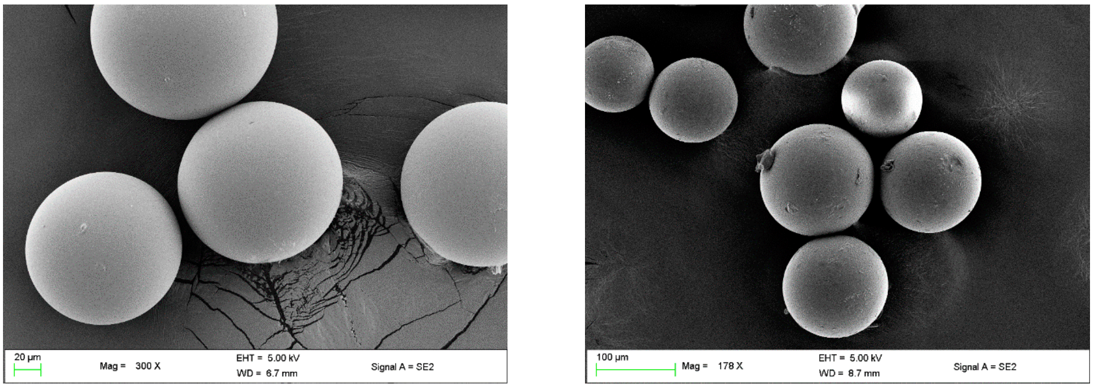


(A) (B)

**Figure 1.** Scanning electron microscope (SEM) images of: (**A**) Aminomethyl (AM) resin; (**B**) 4-methylbenzhydrylamine (MBHA) resin. After the cleavage step and the addition of cyclopentyl methyl ether (CPME) (intact).

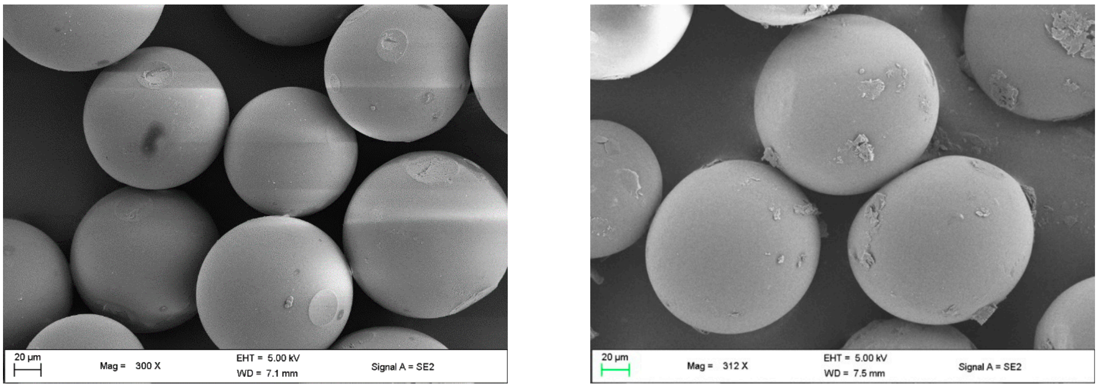


(A) (B)

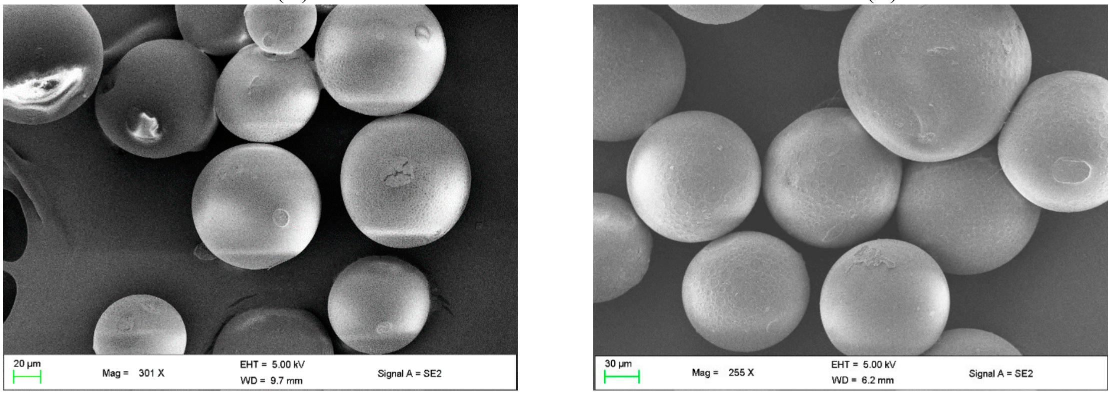


(C) (D)

**Figure 2.** Scanning electron microscope (SEM) images of 2-chlorotrityl chloride (CTC) resin: (**A**) before synthesis starts (native resin); (**B**) before cleavage step (resin plus the synthesized peptide); (**C**) activated native resin (no previous syntheses); (**D**) activated and regenerated resin (after cleaving a peptide).

© 2019 by the authors. Submitted for possible open access publication under the terms and conditions of the Creative Commons Attribution (CC BY) license (http://creativecommons.org/licenses/by/4.0/).
